# The Impact of Concomitant Genomic Alterations on Treatment Outcome for Trastuzumab Therapy in HER2-Positive Gastric Cancer

**DOI:** 10.1038/srep09289

**Published:** 2015-03-19

**Authors:** Ji Yun Lee, Mineui Hong, Seung Tae Kim, Se Hoon Park, Won Ki Kang, Kyoung-Mee Kim, Jeeyun Lee

**Affiliations:** 1 Division of Hematology-Oncology, Department of Medicine, Gastric Cancer Center, Samsung Medical Center, Sungkyunkwan University School of Medicine, 81 Irwon-roGangnam-gu, Seoul 135-710, Korea; 2 Center for Companion Diagnostics, Innovative Cancer Medicine Institute, Samsung Medical Center, Seoul, Korea; 3 Department of Pathology and Translational Genomics, Samsung Medical Center, Sungkyunkwan University School of Medicine, 81 Irwon-roGangnam-gu, Seoul 135-710, Korea

## Abstract

Clinical benefit from trastuzumab and other anti-human epidermal growth factor receptor-2 (HER2) therapies in patients with HER2-positive gastric cancer (GC) remains limited by primary or acquired resistance. We aimed to investigate the impact of concomitant molecular alterations to *HER2* amplification on the clinical outcome of trastuzumab-treated patients. Using immunohistochemistry (IHC), copy number variations (CNVs), and Ion Ampliseq Cancer Panel, we analyzed the status of concomitant alterations in 50 HER2-positive advanced GC patients treated with trastuzumab in combination with other chemotherapeutic agents. The percentage of tumor samples with at least one concomitant alteration was 40% as assessed by IHC, 16% by CNVs, and 64% by Ampliseq sequencing. Median progression-free survival (PFS) was 8.0 months (95% confidence interval, 4.8–11.3). Patients were divided into two subgroups according to PFS values with a cutoff point of 8 months; results show that concomitant genomic alterations do not correlate with trastuzumab response. However, CNVs of *CCNE1* significantly correlated (*p* < 0.05) with a shorter survival time. Our findings indicate that additional alterations implemented for prediction of clinical benefit from HER2-targeting agents in GC remained unclear. Further studies will be needed to elucidate the role of each specific biomarker and to optimize therapeutic approaches.

Gastric cancer (GC) is the fourth most common type of cancer and the second leading cause of cancer-related death in the world^1^. Most patients present with advanced, inoperable or metastatic disease and 5-year survival rates are approximately 30%^2^. Validated chemotherapeutic regimens such as fluoropyrimidine and/or platinum-based therapies failed to improve the prognosis of advanced GC that remains poor, with a median overall survival (OS) being around 1 year^3^,^4^. Therefore, there is an urgent need of targeted-driven approaches toward deregulated molecular signaling pathways in advanced GC such as phosphatidylinositol-4, 5-bisphosphate 3-kinase, catalytic subunit alpha (PIK3CA) pathway or epidermal growth factor receptor (EGFR) pathway. Human epidermal growth factor receptor 2 (HER2) is the first validated treatment target in HER2-positive GC. *HER2* amplification is reported in 7–34% of tumors^5^,^6^. Although anti-HER2 therapy such as trastuzumab confers clinical benefit in GC patients, its efficacy was shown to be unsatisfactory due to primary or acquired resistance^7^,^8^,^9^. The ToGA trial^7^ reported only a modest prolongation of median OS by 2.7 months (from 11.1 months to 13.8 months) with trastuzumab. In addition, TYTAN^8^ and LOGiC^9^ trials failed to demonstrate any survival advantage with another anti-HER2 treatment, lapatinib. To improve clinical outcome of trastuzumab-based chemotherapy in HER2-positive GC, it is necessary to elucidate the role of concomitant genetic alterations in the onset of trastuzumab resistance. This will allow to stratify HER2-positive GC patients according to their sensitivity to anti-HER2 treatments.

Several studies have investigated the onset of trastuzumab resistance in breast cancer therapy. It has been proven that resistance to HER2-targeted therapy may trigger subsequent genetic alterations of receptor tyrosine kinases (RTKs), their downstream signaling targets and alternative pathway activation to compensate for HER2 inhibition^10^,^11^. However, with regard to GC, there are limited preclinical studies demonstrating the possible resistance mechanisms of the HER2 targeting therapies. Based on the assumption that additional oncogenic events co-occurring with *HER2* amplification could affect the response to trastuzumab therapy in metastatic GC, we aimed to further molecularly dissect HER2-positive GC using high throughput sequencing technologies in trastuzumab treated patients.

## Results

### Baseline characteristics

Table 1 shows baseline disease characteristics of patients. The median age of the patients was 60 years and 70% were male. Most of the patients (92%) had good performance status (ECOG, 0–1), 64% presented metastatic GC, and 90% had tubular adenocarcinoma with poorly differentiated cancer (62%). All patients presented HER2-positive tumors with 3+ immunohistochemistry (IHC) scoring as described in the methods section. Patients received trastuzumab plus cisplatin and capecitabine (96%) or trastuzumab plus cisplatin and 5-Fluorouracil (5-FU) (4%).

### Distribution of molecular alterations

**Figure 1:**
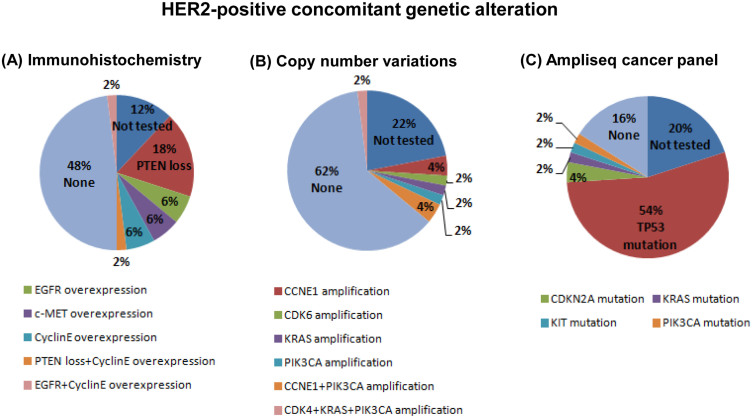
Pie chart summarizing HER2 concomitant genetic alterations as assessed by immunohistochemistry (A), copy number variations (B), and Ampliseq hot spot cancer panel (C).

Figure 1 shows the incidence of specific concomitant genomic alterations with *HER2* amplification as determined by IHC (A), copy number variations (CNVs) (B), and Ion Ampliseq sequencing (C). Twenty patients (40%) presented tumors with at least one co-occurring molecular alteration. In particular, loss of phosphatase and tensin homolog (PTEN) pathway was detected in 20% of the study population while overexpression of EGFR and cyclin E was present in 8% each of the patients; c-MET overexpression was detected in 6% in the patients. Two subjects showed two concomitant molecular alterations in addition to *HER2* amplification, in particular PTEN loss plus cyclin E overexpression and EGFR plus cyclin E overexpression (Figure 1A).

CNVs of 21 genes were determined for 39 out of the 50 tumor samples because there was no archival tissue available for CNV analyses. In addition to *HER2* amplification, 5 genes were concomitantly co-amplified: *CCNE1* (8%), *PIK3CA* (8%), *KRAS* (2%), *CDK4* (2%), and *CDK6* (2%). Of the 21-gene assay, the remaining 16 genes were negative for CNVs (Figure 1B). Three patients presented two or more concomitant CNVs. In particular, CNVs for the *CCNE1* and *PIK3CA* pair and for *CDK4, KRAS* plus *PIK3CA* trio was detected in two and one patients, respectively. A total of 40 out of the 50 HER2-positive tumor samples was sequenced using Ion Ampliseq Cancer Panel to identify hotspot mutation in 50 oncogenes or tumor suppressor genes: *TP53* (54%), *CDKN2A* (4%), *KRAS* (2%), *KIT* (2%), and *PIK3CA* (2%) (Figure 1C).

The clinicopathological parameters including age, sex, ECOG PS, extent of disease, tumor location, differentiation, and morphology showed no significant associations with co-occurring genetic alterations (Supplemental Table 1). In this patient cohort, only female patients demonstrated significantly higher EGFR overexpression than male patients (*p* = 0.039) (Supplementary Table 1).

### Correlation between concomitant alterations and clinical outcome of HER2-targeted therapy

**Figure 2:**
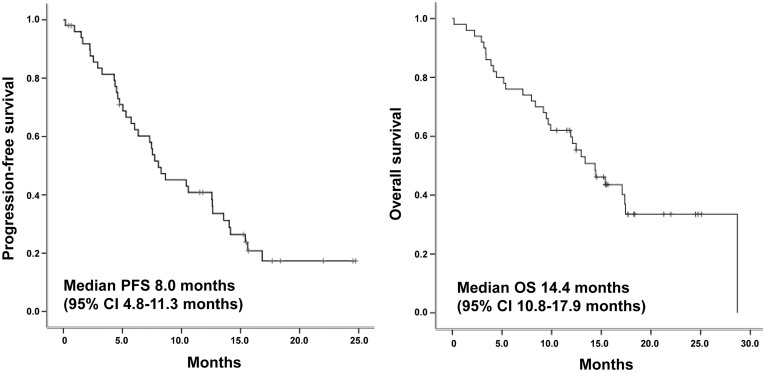
Kaplan-Meier survival curves of study population.

With median number of 8 cycles (range, 1–32), overall tumor response rate was as follows: complete response (CR, 2%), partial response (PR, 52%), stable disease (SD, 28%), progressive disease (PD, 4%), missing (14%). There was no clinicopathologic feature or additional alteration associated with objective response (supplementary Table 2). Median progression-free survival (PFS) and OS were 8.0 months (95% CI, 4.8–11.3) and 14.4 months (95% CI, 10.8–17.9), respectively (Figure 2). Patients were dichotomized into < or ≥ median PFS groups (<8 months vs. ≥8 months) based median PFS derived from this study cohort. Among clinicopathologic variables, tumor differentiation was the only predictive factor for poor response to trastuzumab-based chemotherapy (*p* = 0.024). Notably, 4 patients with HER2-positive GC patients and concomitant amplification of *CCNE1* demonstrated a significantly shorter PFS (PFS < 8 months, *p* = 0.047) (Table 2). The overall tumor responses of these patients were as follows: SD (2 patients), PR (one patient), CR (one patient). A logistic regression analysis was performed in order to further evaluate the significant prognostic value of all the additional genetic alterations that might positively affect the PFS of trastuzumab treatment in HER2-positive GC. Results failed to identify any independent factor for the prediction of trastuzumab response. (PTEN loss, *p* = 0.627; EGFR overexpression, *p* = 0.731; c-MET overexpression, *p* = 0.861; cyclin E overexpression, *p* = 0.147).

## Discussion

Targeted anti-HER2 therapy has failed to substantially ameliorate the clinical outcome of patients affected by HER2-positive GC. In the attempt to further investigate how concomitant molecular alterations influence response to trastuzumab based chemotherapy, we studied the expression status of four biomarkers: PTEN, EGFR, c-MET ad cyclin E using IHC, CNV array and NGS sequencing. In our previous study, we profiled 434 GC patients using a proteomic approach and demonstrated a concomitant activation of HER2 with other RTKs in a substantial number of GCs^12^. In addition, we also identified that a small percentage of HER2-positive GC patients harbor concomitant c-MET overexpression who pursued more aggressive clinical course then HER2-positive alone GC patients^13^. In line with this, pre-existing secondary molecular alterations were identified in 54.8% of *HER2*-amplified gastroesophageal (GE) adenocarcinomas in vitro and some of these alterations conferred resistance to HER2-directed therapy^14^.

Concomitant and recurrent focal amplifications were identified in HER2-positive GE tumors at the loci of *CCNE1, CDK6, EGFR, MET* and *MYC* based on previous TCGA study. In addition, the mutational profile of the 42 *HER2*-amplified GE tumors revealed concomitant *PIK3CA* mutations in 19.0% of cases. In our patient cohort, there was only one patient with *PIK3CA* L1047R mutation and one with *PIK3CA* E542K, known activating mutations in several cancer types^15^. Therefore, it is inconclusive from our study regarding chemo-responsiveness to trastuzumab in *HER2*-amplified GC tumors with *PIK3CA* mutation. *In vitro* cell line data, however, demonstrated that *PIK3CA* mutations significantly reduced sensitivity to HER2-targeting agents in *HER2*-amplified GE cells^14^.

Phosphatidylinositol-3-kinase (PI3K)-Akt-mammalian target of rapamycin (mTOR) cascade is a pathway downstream of HER2 and phosphatase and tensin homolog (PTEN) antagonizes the function of PI3K^16^,^17^. PI3K pathway activation, defined as PTEN loss and/or *PIK3CA* mutation, was associated with a poor response to trastuzumab and a shorter survival time in breast cancer^18^,^19^. Furthermore, *PIK3CA* mutation and PTEN loss were observed in 4–36%^20^,^21^,^22^ and 17–52%^20^,^22^,^23^ of GC tissues. There are limited data published on the role of the PTEN loss in trastuzumab therapy for HER2-positive advanced GC. A recent study highlighted a correlation between PTEN loss, observed in 83% of HER2-positive cases, and a shortened survival time^24^. In our study, PTEN loss and *PIK3CA* E542K hotspot mutation were detected in 20% and 2% of the tumor samples, respectively. However, we did not observe a significant correlation between PTEN loss and response to trastuzumab therapy in this patient cohort.

With regard to trastuzumab response and expression status of *EGFR* and *c-MET* genes, it has been reported that overexpression of both biomarkers can contribute to the onset of resistance toward trastuzumab therapy in breast cancer^25^,^26^. These studies suggest that HER2-positive patients harboring such genetic alterations might benefit from additional EGFR and/or c-MET targeted therapy. The criteria for overexpression of EGFR (6%) and c-MET protein (6%) were described in our previous study^13^. However, in the present study, due to a small sample size, the impact of EGFR overexpression could not be analyzed further.

The frequent amplifications of the cell cycle mediators, *CDK6, CCND1* and *CCNE1* were observed in *HER2*-amplified GE cancer^14^. *In vitro* study of MKN7, which harbors pre-existing amplifications of both *CCNE1* and *HER2* demonstrated that the cell line was relatively insensitive to lapatinib or the CDK2 inhibitor AZD5438, but sensitive to combination treatment. We have previously shown that Cyclin E1 (encoded by *CCNE1* gene) and HER2 and its associated CDK2 are essential for cellular progression through the G1 phase of the cell cycle and initiation of DNA replication^14^. An excess of cyclin E may therefore render cells independent from trastuzumab-mediated cell cycle arrest^27^. *CCNE1* amplification is reported in up to 24% of HER2-positive breast cancers and it has been suggested to play a role during trastuzumab resistance in breast cancer^28^. In the present study, 10% of patients showed CNVs of CCNE1 and this significantly correlated with poor PFS following trastuzumab-based chemotherapy. Although CCNE1 amplification was significantly correlated with high expression of cyclin E protein (p < 0.000, rho correlation efficient 0.623), there was no correlation between clinical outcome and cyclin E expression status. Taken together, all four patients with concomitant *CCNE1* amplification and *HER2* amplification progressed shortly after trastuzumab-based chemotherapy. Hence, in this subset of patients, combination therapy should be tested in the context of clinical trials.

In this study, we comprehensively interrogated the existence of concomitant genomic alterations of *HER2*-amplified GC tumors in trastuzumab-treated GC patient cohort. All tumors were procured before the start of HER2-directed therapy. Although limited by small sample size, we identified that *HER2*-amplified GC patients do have diverse pattern of various concurrent molecular events. Clinically, *CCNE1* amplification was the only marker which was a significant adverse predictor to trastuzumab response. Nevertheless, given the low incidence of concomitant *MET* amplification as well as *EGFR* amplification in *HER2*-amplified patient cohort with trastuzumab therapy, combined effort should be sought to draw statistically meaningful predictive molecular markers. Lastly, dual inhibition of *CCNE1* amplification and HER2-directed therapy may be considered as a clinical trial option in *CCNE1*-amplified, HER2-positive GC patients. Development of inhibitors targeting multiple receptors or common downstream signaling proteins merits further investigation.

## Methods

### Study population

Primary tumor tissue was available from 50 patients with HER2-positive advanced GC who had received trastuzumab-based chemotherapy between 2009 and 2012. All tissue specimens were obtained before treatment. Tumor samples were analyzed using IHC (PTEN, EGFR, c-MET, and cyclin E), copy number variations (CNVs: CCNE1, CDK4, CDK6, EGFR, ERBB2, KRAS, MET, MDM2, and PIK3CA) and Ion Torrent Ampliseq Cancer Panel v2.0.

Data regarding patient demographics, pathological classification, treatment response, progression-free survival (PFS), and OS were obtained retrospectively by reviewing medical records. All patients treated with trastuzumab plus chemotherapy (cisplatin plus capecitabine or 5-fluorouracil). Capecitabine 1000 mg/m2 was given orally twice a day for 14 days followed by a 1-week rest, or 5-fluorouracil (5-FU) 800 mg/m2 per day was given by continuous intravenous infusion on days 1–5 of each cycle. Cisplatin 80 mg/m2 on day 1 was given by intravenous infusion. Trastuzumab was given by intravenous infusion at a dose of 8 mg/kg on day 1 of the first cycle, followed by 6 mg/kg every 3 weeks until disease progression, unacceptable toxicity. Treatment response was evaluated using a spiral computed tomography (CT) scan and the Response Evaluation Criteria in Solid Tumors (RECIST) version 1.1^29^. Written informed consent form, approved by the Institutional Review Board, was obtained from all patients before the study. All experimental procedures were carried out in accordance with guidelines approved by Samsung Medical Center.

### Immunohistochemistry for HER2, PTEN, EGFR, c-MET, and cyclin E

All the tissue samples were obtained before chemotherapy and fixed in buffered 10% formalin and embedded in paraffin. Primary antibodies included HER2 (4B5, Ventana Medical Systems, Tucson, AZ, USA), PTEN (Y184; Abcam, Cambridge, UK), EGFR (anti-NCL-L-EGFR-384, Novocastra/Vision Biosystems, Newcastle, UK), CONFIRM anti-Total c-MET (SP44; Ventana Medical Systems, Tucson, AZ, USA), and cyclin E (HE12; Thermo Scientific, Leicestershire, UK). The BenchMark XT automated slide processing system (Ventana Medical Systems) was used according to the manufacturer's protocol. HER2 status was scored according to gastric criteria proposed by Rüschoff et al^30^. The intensity of immunostaining for EGFR and c-MET was graded as 0 (negative), 1+ (weak), 2+ (moderate), and 3+ (strong) in >10% of tumor cells and only 2+ or 3+ were interpreted as being positive^13^. PTEN expression was analyzed at both cytoplasmic and nuclear levels. Low level of cyclin E was defined when less than 50% of tumor cell nuclei were stained^31^.

### Fluorescence in situ hybridization (FISH) for *HER2* amplification

FISH was performed as previously described using dual-color DNA-specific probes from PathVysion™ (LSI® HER2 Spectrum Orange™ and CEP17 Spectrum Green™; Abbott, San Francisco, CA, USA). The *HER2* gene was considered amplified when the FISH signal ratio of *HER2*/CEP17 was ≥2.0. Tumor samples were classified as HER2-positive when IHC score was 3+ or 2+ in combination with *HER2* gene amplification confirmed by FISH^32^.

### DNA preparation

DNA was extracted from 3- to 5-μm-thick formalin-fixed paraffin-embedded (FFPE) tissue samples after deparaffinization with xylene and by using the QIAamp DNA Mini Kit (Qiagen) according to the manufacturer's instructions.

### NanoString nCounter assay

For detection of CNVs, a panel of 21 gene probes including *CCNE1, CDK4, CDK6, EGFR, ERBB2, FGFR2, KRAS, MDM2,* and *PIK3CA* were designed using NanoString nCounter technology and subsequently analyzed on the NanoString nCounter platform (NanoString Technologies, Seattle, WA, USA)^33^,^34^. Three probes were designed for each gene. Each assay contained six positive dsDNA control probes, 8 negative control probes, and 10 invariant control probes (INVs) designed for autosomal genomic regions predicted not to contain common CNVs.

The NanoString nCounter assay was performed according to NanoString's standard protocol. Briefly, 300 ng of fragmented genomic DNA per sample was hybridized with the capture and reporter probes in a total volume of 30 μl and incubated overnight at 65°C. Target and probe complexes were washed and immobilized in the cartridge. Genomic DNA was fragmented into small pieces (200–800 bp) and subsequently denatured to produce single strands. Custom CNV CodeSet was then hybridized to the fragmented denatured DNA sample in a single multiplexed reaction. Hybridized DNA-CodeSet complexes were purified using a fully automated nCounter prep station, and reporters were counted using the nCounter digital analyzer. Data were normalized to the INVs and to positive and negative controls in each hybridization reaction. Finally, data analysis was performed using nSolver software.

Copy number was determined by averaging over three probes per region. If the average copy number was below 1.4, the gene was considered as one copy; if between 1.5 and 2.4, considered as two copies; and if between 2.5 and 3.4, considered as three copies, according to the manufacturer's protocol.

### Ion Torrent PGM library preparation and sequencing

An Ion Torrent adapter-ligated library was prepared following the manufacturer's Ion AmpliSeq Library Kit 2.0 protocol (Life Technologies, Part #4475345 Rev. A). A total number of 2,855 mutations in 50 commonly mutated oncogenes and tumor suppressor genes were examined. First, 200 ng of DNA from each of tumor samples underwent single-tube, multiplex PCR amplification using the Ion AmpliSeq Cancer Primer Pool and the Ion AmpliSeq Kit reagents (Life Technologies). Treatment of the resulting amplicons with FuPa reagent partially digested the primers and phosphorylated the amplicons. The phosphorylated amplicons were ligated to Ion Adapters and purified. For barcoded library preparation, barcoded adapters were substituted from the Ion Xpress™ Barcode Adapters 1–96 Kit for the non-barcoded adapter mix supplied in the Ion AmpliSeq™ Library Kit. The ligated DNA underwent nick-translation and amplification to complete the linkage between adapters and amplicons and to generate sufficient material for downstream template preparation. Two rounds of Agencourt® AMPure® XP Reagent binding at 0.6 and 1.2 bead-to-sample volume ratios removed input DNA and unincorporated primers from the amplicons. The final library molecules were 125~300 bp in size. Libraries were then transferred to the Ion OneTouch™ System for automated template preparation. Sequencing was performed on the Ion PGM™ sequencer according to the manufacturer's instructions. IonTorrent Software was used for automated data analysis. An Ion Sequencing Kit v2.0 was used for sequencing reactions, following the recommended protocol (Life Technologies, Part Number 4469714 Rev. B). *KRAS* and *PIK3CA* mutations were confirmed by cobas® *KRAS* mutation test kit and cobas® 4800 System *PIK3CA* mutation test kit (Roche Diagnostics, Rotkreuz, Switzerland) for the detection mutations in exons 1, 4, 7, 9 and 20.

### Statistical analysis

The cohort was analyzed on an intention-to-treat (ITT) basis. OS was defined as the time (days) from the date of trastuzumab treatment initiation in combination with chemotherapy until the date of death from any cause. PFS was defined as the time (days) from the date of trastuzumab treatment initiation in combination with chemotherapy until the date of disease progression or death from any cause. The survival curves were derived by the Kaplan-Meier methods for all patients (n = 50). In order to identify the predictive value of additional alterations for HER2-targeted therapy, patients' cohort was dichotomized in terms of short/long PFS values (<8 months *vs.* ≥8 months); cutoff value was chosen taking into account the median PFS (8 months) in our study population. The associations between categorical variables and each dichotomized group were analyzed by chi-square test or Fisher's exact test. Two-sided *p* values of less than 0.05 were considered significant. Statistical tests were performed using SPSS 21.0 software (IBM Corporation, New York, NY, USA).

## Supplementary Material

Supplementary Information Supplemental file

## Figures and Tables

**Table 1:** Baseline characteristics of the patients

Variable	Patients (n = 50)
Age-year	
Median	60
Range	30–78
Sex, no. (%)	
Male	35 (70)
Female	15 (30)
ECOG performance status, no. (%)	
0–1	46 (92)
2	4 (8)
Extent of disease, no. (%)	
Locally advanced	9 (18)
Metastatic	32 (64)
Postoperative relapse	9 (18)
Tumor location, no. (%)	
Antrum	26 (52)
Fundus/body	19 (38)
Cardia/GEJ	5 (10)
Tumor histology, no. (%)	
Tubular	44 (90)
Signet ring cell	3 (6)
Papillary	2 (4)
Tumor differentiation, no. (%)	
Well	0
Moderate	19 (38)
Poor	31 (62)
First line of chemotherapy, no. (%)	
XP/Trastuzumab	48 (96)
FP/Trastuzumab	2 (4)

ECOG, Eastern Cooperative Oncology Group performance status; GEJ, gastroesophageal junction; XP, capecitabine and cisplatin; FP, fluorouracil and cisplatin.

**Table 2:** Subgroup analysis according to progression-free survival of patients

Characteristics	PFS < 8 months	PFS ≥ 8 months	*p* value
**Clinical variables**			
Age, n (%)			0.671
Age < 60	10 (42)	11 (48)	
Age ≥ 60	14 (58)	12 (52)	
Sex, n (%)			0.266
Male	20 (77)	15 (63)	
Female	6 (23)	9 (37)	
ECOG performance status, n (%)			0.111
0–1	22 (85)	24 (100)	
2	4 (15)	0	
Extent of disease, n (%)			1.000
Locally advanced	3 (12)	6 (25)	
Metastatic	20 (77)	12 (50)	
Postoperative relapse	3 (11)	6 (25)	
Tumor location, n (%)			0.099
Antrum	10 (39)	16 (67)	
Body/fundus	13 (50)	6 (25)	
Cardia/GEJ	3 (11)	2 (8)	
Histology, n (%)			0.721
Tubular	23 (92)	21 (88)	
Signet ring cell	1 (4)	2 (8)	
Papillary	1 (4)	1 (4)	
Differentiation			0.024
Moderate	6 (23)	13 (54)	
Poor	20 (77)	11 (46)	
**Immunohistochemistry**			
PTEN loss			0.724
Absent	17 (74)	17 (81)	
Present	6 (26)	4 (19)	
EGFR overexpression			0.658
Absent	21 (91)	18 (86)	
Present	2 (9)	3 (14)	
c-MET overexpression			0.605
Absent	19 (95)	17 (90)	
Present	1 (5)	2 (10)	
Cyclin E overexpression			0.172
Absent	15 (79)	20 (95)	
Present	4 (21)	1 (5)	
**Copy number variations**			
CCNE1 amplification			0.047
Absent	15 (79)	20 (100)	
Present	4 (21)	0	
PIK3CA amplification			1.000
Absent	17 (90)	18 (90)	
Present	2 (10)	2 (10)	
**Ampliseq cancer panel**			
TP 53 mutation			0.577
Absent	6 (29)	7 (37)	
Present	15 (71)	12 (63)	

ECOG, Eastern Cooperative Oncology Group performance status; GEJ, gastroesophageal junction.
