# Supplementary material for: The Impact of Concomitant Genomic Alterations on Treatment Outcome for Trastuzumab Therapy in HER2-Positive Gastric Cancer
Source: Sci Rep. 2015 Mar 19;5:9289. doi: 10.1038/srep09289 (PMC5380124; doi:10.1038/srep09289)
Supplement: Supplementary Information — Supplemental file [file srep09289-s1.doc]

**The Impact of Concomitant Genomic Alterations on Treatment Outcome for Trastuzumab Therapy in HER2-Positive Gastric Cancer**

Ji Yun Lee,¹^*^ Mineui Hong,^2,3*^ Seung Tae Kim,^1^ Se Hoon Park,^1^ Won Ki Kang,^1^
Kyoung-Mee Kim^2,3†^ & Jeeyun Lee^1†^

^1^Division of Hematology-Oncology, Department of Medicine, Gastric Cancer Center, Samsung Medical Center, Sungkyunkwan University School of Medicine, 81 Irwon-ro, Gangnam-gu, Seoul 135-710, Korea, ^2^Center for Companion Diagnostics, Innovative Cancer Medicine Institute, Samsung Medical Center, Seoul, Korea, ^3^Department of Pathology and Translational Genomics, Samsung Medical Center, Sungkyunkwan University School of Medicine, 81 Irwon-ro, Gangnam-gu, Seoul 135-710, Korea

**Equally contributing authors.*

**Supplemental Table 1 (A) PTEN, EGFR, c-MET, and Cyclin E protein expression and cliniopathological features of patients**

|  | No. | PTEN  (n=34) | *p*  value | EGFR  (n=5) | *p*  value | c-MET  (n=3) | *p*  value | Cyclin E  (n=5) | | *p*  value |
| --- | --- | --- | --- | --- | --- | --- | --- | --- | --- | --- |
| Age, n (%) |  |  | 0.489 |  |  |  |  |  |  | |
| <60 | 18 | 15 (83) |  | 1 (6) | 0.634 | 1 (6) | 1.000 | 0 | 0.061 | |
| ≥60 | 26 | 19 (73) |  | 4 (15) |  | 2 (9) |  | 5 (22) |  | |
| Sex, n (%) |  |  | 0.714 |  |  |  |  |  |  | |
| Male | 29 | 23 (29) |  | 1 (3) | 0.039* | 2 (7) | 1.000 | 5 (19) | 0.143 | |
| Female | 15 | 11 (73) |  | 4 (27) |  | 1 (8) |  | 0 |  | |
| ECOG PS, n (%) | |  |  |  |  |  |  |  |  | |
| 0-1 | 42 | 32 (76) | 1.000 | 4 (10) | 0.217 | 3 (8) | 1.000 | 4 (11) | 0.237 | |
| 2 | 2 | 2 (100) |  | 1 (50) |  | 0 |  | 1 (50) |  | |
| Extent, n (%) | |  |  |  |  |  |  |  |  | |
| Others† | 17 | 15 (88) | 0.271 | 2 (12) | 1.000 | 0 | 0.271 | 3 (21) | 0.322 | |
| Meta | 27 | 19 (70) |  | 3 (11) |  | 3 (13) |  | 2 (8) |  | |
| Location, n (%) | |  |  |  |  |  |  |  |  | |
| Antrum | 21 | 16 (76) | 0.849 | 1 (5) | 0.207 | 2 (11) | 0.468 | 4 (20) | 0.155 | |
| Body/fundus | 18 | 14 (78) |  | 13 (17) |  | 1 (6) |  | 1 (6) |  | |
| Cardia/GEJ | 5 | 4 (80) |  | 1 (20) |  | 0 |  | 0 |  | |
| Differentiation, n (%) | | |  |  |  |  |  |  |  | |
| Moderate | 17 | 12 (71) | 0.401 | 3 (18) | 0.359 | 1 (7) | 1.000 | 3 (18) | 0.634 | |
| Poor | 27 | 22 (82) |  | 2 (8) |  | 2 (8) |  | 2 (9) |  | |
| Morphology, n (%) | |  |  |  |  |  |  |  |  | |
| Tubular | 38 | 30 (79) | 0.307 | 5 (13) | 0.424 | 3 (9) | 0.566 | 4 (11) | 0.283 | |
| Signet | 3 | 2 (67) |  | 0 |  | 0 |  | 0 |  | |
| Papillary | 2 | 1 (50) |  | 0 |  | 0 |  | 1 (50) |  | |

*p <0.05 †postoperative relapse or locally advanced.

ECOG, Eastern Cooperative Oncology Group performance status; GEJ, gastroesophageal junction

**Supplemental Table 1 (B) *CCNE1* and *PIK3CA* copy number variations, *TP53* Ampliseq sequencing and cliniopathological features of patients**

|  | No. | *CCNE1*  (n=4) | *p*  value | *PIK3CA*  (n=4) | *p*  value | No. | *TP53*  (n=27) | *p*  value |
| --- | --- | --- | --- | --- | --- | --- | --- | --- |
| Age, n (%) | |  |  |  |  |  |  |  |
| < 60 | 15 | 1 (7) | 1.000 | 1 (7) | 1.000 | 16 | 12 (75) | 0.503 |
| ≥ 60 | 24 | 3 (13) |  | 3 (13) |  | 24 | 15 (63) |  |
| Sex, n (%) |  |  |  |  |  |  |  |  |
| Male | 26 | 4 (15) | 0.281 | 2 (8) | 0.589 | 27 | 18 (67) | 1.000 |
| Female | 13 | 0 |  | 2 (15) |  | 13 | 9 (69) |  |
| ECOG PS, n (%) | |  |  |  |  |  |  |  |
| 0-1 | 37 | 4 (11) | 1.000 | 4 (11) | 1.000 | 38 | 26 (68) | 1.000 |
| 2 | 2 | 0 |  | 0 (0) |  | 2 | 1 (50) |  |
| Extent, n (%) | |  |  |  |  |  |  |  |
| Others† | 17 | 1 (6) | 0.618 | 1 (6) | 0.618 | 16 | 11 (69) | 0.890 |
| Meta | 22 | 3 (14) |  | 3 (14) |  | 24 | 16 (67) |  |
| Location, n (%) | |  |  |  |  |  |  |  |
| Antrum | 19 | 3 (16) | 0.252 | 2 (11) | 0.718 | 18 | 10 (56) | 0.079 |
| Body/fundus | 16 | 1 (6) |  | 2 (13) |  | 18 | 13 (72) |  |
| Cardia/GEJ | 4 | 0 |  | 0 |  | 4 | 4 (100) |  |
| Differentiation, n (%) | | |  |  |  |  |  |  |
| Moderate | 14 | 1 (7) | 1.000 | 2 (14) | 0.609 | 14 | 8 (57) | 0.305 |
| Poor | 25 | 2 (12) |  | 2 (8) |  | 26 | 19 (73) |  |
| Morphology, n (%) | | |  |  |  |  |  |  |
| Tubular | 34 | 4 (12) | 0.502 | 3 (9) | 0.545 | 35 | 22 (63) | 0.166 |
| Signet | 3 | 0 |  | 1 (33) |  | 3 | 3 (100) |  |
| Papillary | 1 | 0 |  | 0 |  | 1 | 1 (100) |  |

†postoperative relapse or locally advanced. GEJ: gastroesophageal junction.

**Supplemental Table 2 Concomitant alterations and object response**

| Characteristics | No. | Object response  (CR+PR) | *p* value |
| --- | --- | --- | --- |
| PTEN loss by IHC, n (%) |  |  |  |
| Absent | 29 | 18 (62) | 0.685 |
| Present | 8 | 6 (75) |  |
| EGFR overexpression by IHC, n (%) | | |  |
| Absent | 33 | 20 (61) | 0.276 |
| Present | 4 | 4 (100) |  |
| c-MET overexpression by IHC, n (%) | | |  |
| Absent | 31 | 19 (61) | 0.537 |
| Present | 3 | 3 (100) |  |
| Cyclin E overexpression by IHC, n (%) | | |  |
| Absent | 30 | 20 (67) | 0.602 |
| Present | 4 | 2 (50) |  |
| *CCNE1* by CNVs, n (%) |  |  |  |
| Absent | 30 | 19 (63) | 1.000 |
| Present | 4 | 3 (75) |  |
| *PIK3CA* by CNVs, n (%) |  |  |  |
| Absent | 30 | 20 (67) | 0.602 |
| Present | 4 | 2 (50) |  |
| *TP 53* mutation by Ampliseq, n (%) | | |  |
| Absent | 11 | 5 (50) | 0.176 |
| Present | 23 | 16 (70) |  |
